# Clinical features and surgical outcomes of high grade pleomorphic xanthoastrocytomas: a single-center experience with a systematic review

**DOI:** 10.3389/fonc.2023.1193611

**Published:** 2023-06-28

**Authors:** Pengcheng Zuo, Tian Li, Tao Sun, Wenhao Wu, Yujin Wang, Mingxin Zhang, Zhen Wu, Junting Zhang, Liwei Zhang

**Affiliations:** ^1^Department of Neurosurgery, Beijing Tiantan Hospital, Capital Medical University, Beijing, China; ^2^China National Clinical Research Center for Neurological Diseases, Beijing, China

**Keywords:** high grade, pleomorphic xanthoastrocytoma, gross-total resection, radiotherapy, chemotherapy

## Abstract

**Purpose:**

High grade pleomorphic xanthoastrocytomas (HGPXAs) are very rare and their management and prognostic outcomes remain unclear. To better understand the disease, we aimed to evaluate the risk factors for progression-free survival (PFS) and overall survival (OS), and propose a treatment protocol based on cases from our institute and cases from the literature.

**Methods:**

The authors reviewed the clinical data of 26 patients with HGPXAs who underwent surgical treatment in Department of Neurosurgery of Beijing Tiantan Hospital between August 2014 and September 2021. We also searched the PubMed database using the keywords “anaplastic” combined with “pleomorphic xanthoastrocytoma(s)” between January 1997 and October 2022. Risk factors for PFS and OS were evaluated in the pooled cases.

**Results:**

The authors’ cohort included 11 males and 15 females with a mean age of 36.7 ± 20.3 years (range: 5.5-71 years). Gross-total resection (GTR) and non-GTR were achieved in 17 (65.4%) and 9 (34.6%) patients, respectively. Radiotherapy and chemotherapy were administered to 22 and 20 patients, respectively. After a mean follow-up of 20.5 ± 21.2 months (range: 0.5-78.1 months), 7 patients suffered tumor recurrence and 6 patients died with a mean OS time of 19.4 ± 10.8 months (range: 8-36 months). In the literature between January 1997 and October 2022, 56 cases of HGPXAs were identified in 29 males and 27 females with a mean age of 29.6 ± 19.6 years (range; 4-74 years). Among them, 24 (44.4%) patients achieved GTR. Radiotherapy and chemotherapy was administered to 31 (62%) patients and 23 (46%) patients, respectively. After a median follow-up of 31.4 ± 35.3 months (range: 0.75-144 months), the mortality and recurrence rates were 32.5% (13/40) and 70% (28/40), respectively. Multivariate Cox regression model demonstrated that non-GTR (HR 0.380, 95% CI 0.174-0.831, p=0.015), age≥30 (HR 2.620, 95% CI 1.183-5.804, p=0.018), no RT (HR 0.334,95% CI 0.150-0.744, p=0.007) and no CT (HR 0.422, 95% CI 0.184-0.967, p=0.042) were negative prognostic factors for PFS. Non-GTR (HR 0.126, 95% CI 0.037-0.422, p=0.001), secondary HGPXAs (HR 7.567, 95% CI 2.221-25.781, p=0.001), age≥30 (HR 3.568, 95% CI 1.190-10.694, p=0.023) and no RT (HR 0.223,95% CI 0.073-0.681, p=0.008) were risk factors for OS.

**Conclusion:**

High grade pleomorphic xanthoastrocytomas are very rare brain tumors. Children and younger adults have better clinical outcome than elderly patients. Secondary HGPXAs had worse OS than primary HGPXAs. Complete surgical excision plus RT and CT is recommended for this entity. The frequency of BRAF mutations in HGPXAs is 47.5% (19/40) in this study, however, we do not find the connections between BRAF mutations and clinical outcomes. Future studies with larger cohorts are necessary to verify our findings.

## Introduction

Pleomorphic xanthoastrocytomas (PXAs) are rare brain tumors which often occur in children and young adults (1–3). In 1999, Giannini et al. defined ‘PXA with anaplastic features’ as PXA exhibiting increased mitotic activity, >5/10 HPF mitotic figures with or without accompanying necrosis (4). ‘PXA with anaplastic features’ are labelled as anaplastic pleomorphic xanthoastrocytoma, according to the 2016 World Health Organization Classification of Tumors of the Central Nervous System (3). In the fifth edition of the WHO Classification of Tumors of the Central Nervous System, the term “anaplastic pleomorphic xanthoastrocytoma” is no longer listed, however, according to histopathological features of PXA, this entity can still be classified into WHO grade 2 or WHO grade 3 tumors (5). The clinical prognosis of patients with PXAs is usually satisfactory, with a 5-year survival rate of 80% (6). However, patients with HGPXAs have a worse clinical outcome than patients with PXAs (7). Due to the rarity of the tumor, their management and prognostic outcomes remain unclear. In this study, we reported 26 cases with HGPXAs in our institute and performed a pooled analysis of individual data (including cases from our institute and 56 cases from the literature) to propose a treatment protocol.

## Methods

We performed a retrospective analysis of 26 cases of high grade pleomorphic xanthoastrocytomas (HGPXAs). All the patients accepted surgery in Beijing Tiantan Hospital between August 2014 and September 2021. The following clinical data were included: age, sex, imaging characteristics, extent of tumor resection, histopathological results, treatment protocol and outcomes. Pre- and postoperative MRI images were performed to evaluate the extent of tumor excision, which was defined as gross total resection (GTR) and non-GTR. The follow-up was performed by telephone interview every six months. This research was approved by the Beijing Tiantan Hospital Research Ethics Committee. The pathological diagnosis of HGPXAs was confirmed by the Department of Neuropathology at Beijing Neurosurgical Institute according to the 2021 World Health Organization Classification of Tumors of the Central Nervous System. Histopathological examination showed malignant glial component with numerous mitoses. Eosinophilic granules and ribosome-lamellar complexes can be observed under the light microscope, which are characteristic features of PXA. All cases exhibiting increased mitotic activity, >5/10 HPF mitotic figures with or without accompanying necrosis. The mutation status of BRAF, IDH, and TERT promoter, the methylation status of the MGMT promoter, and the co-deletion status of 1p/19q were also assessed in some of our cases.For the pooled analysis of HGPXAs, we performed a search in the PubMed database between January 1997 and October 2022. The keyword used was “anaplastic” combined with “pleomorphic xanthoastrocytoma(s)” and a total of 56 cases were included. All cases were pathologically diagnosed as “anaplastic pleomorphic xanthoastrocytoma”, which were classified into WHO grade 3 PXAs.

Cox regression models were used to evaluate variables and their association with PFS and OS. The Kaplan-Meier method was used to determine the OS and PFS differences with p-values calculated from the log-rank test. Analyses were performed using SPSS Statistical Package software with the significant set at p < 0.05.

## Results

### Cases from our institute

The author’s cohort included 11 males and 15 females with an average age of 36.7 ± 20.3 years (range: 5.5-71 years). Preoperative seizure was observed in 8 (30.8%) patients. 22 patients experienced primary HGPXAs, 4 patients suffered malignant transformation of previous PXAs. The lesion locations included temporal lobe (n=13), frontal lobe (n=3), occipital lobe (n=2), parietal lobe (n=2), frontal-parietal lobe (n=2), parietal-occipital (n=1), lateral ventricle (n=1), brainstem (n=1), cerebellopontine angle area (n=1). The morphology was classified as solid (n=16) and cystic-solid (n=10). MRI scans showed that peritumoral edema was significant in 21 (80.8%) patients and enhancement was observed in 25 (96.2%) patients. All patients accepted surgical treatment in our hospital. Gross-total resection (GTR) and non-GTR were achieved in 17 (65.4%) and 9 (34.6%) patients, respectively. Combined radiotherapy and chemotherapy was administered to 19 (73.1%) patients, radiotherapy alone was administered to 3 (11.5%) patients, chemotherapy alone was administered to 1 (3.9%) patient and 3 (11.5%) patients did not undergo any adjuvant therapy. After a median follow-up of 20.5 ± 21.2 months (range: 0.5-78.1 months), 7 patients suffered tumor recurrence and 6 patients died with a mean OS time of 19.4 ± 10.8 months (range: 8-36 months). Immunohistochemistry of this series showed a more or less consistent positivity for GFAP, S100, Olig-2 and CD34 with a significant Ki67 expression (ranged from 3.5% to 60%). In our study, 40% (6/15) of the patients exhibited the BRAF V600E mutation, while none of the available patients (13 patients) had IDH mutations. Additionally, we identified TERT promoter mutation in 3 out of the 13 cases examined. Testing the methylation status of the MGMT promoter in 13 patients revealed that 3 patients had MGMT promoter methylation. Furthermore, we examined the co-deletion status of 1p/19q in 15 patients, and none of them exhibited co-deletion of 1p/19q (**Table 1**).

**Table 1:** General characteristics of 26 cases in our institute.

Case	Sex	Age (years)	Seizure	Site	Primary & Secondary	Peritumoral edema	Solid/cystic-solid	Enhancement	Treatment	BRAF	Ki-67 (%)	IDH1	MGMT promoter	1p/19q codeletion	TERT promoter mutation	PFS (months)	Follow-up time (months)	Outcome	Recurrence
1	M	65	No	Frontal-parietal	Primary	Yes	cystic-solid	Yes	Non-GTR	NA	3.5	NA	NA	NA	NA	11.0	18.0	Dead	Yes
2	F	33	Yes	Temporal	Secondary	Yes	cystic-solid	Yes	GTR+RT	NA	60	-	unmethylated	-	NA	12.0	20.0	Dead	Yes
3	F	8	Yes	Temporal	Primary	Yes	solid	Yes	GTR+RT	NA	NA	NA	NA	NA	NA	78.1	78.1	Alive	No
4	F	5.5	Yes	Temporal	Primary	No	solid	Yes	GTR+RT	NA	NA	NA	NA	NA	NA	69.5	69.5	Alive	No
5	F	9	No	Parietal-occipital	Primary	Yes	cystic-solid	Yes	GTR	NA	NA	NA	NA	NA	NA	68.3	68.3	Alive	No
6	M	24	No	Lateral ventricle	Primary	Yes	solid	Yes	Non-GTR+RT+CT	NA	NA	NA	NA	NA	NA	26.0	36.0	Dead	Yes
7	F	45	No	Temporal	Primary	Yes	solid	Yes	Non-GTR+CT	NA	NA	NA	NA	NA	NA	6.0	8.0	Dead	Yes
8	F	37	Yes	occipital	Primary	Yes	cystic-solid	Yes	GTR+RT+CT	NA	NA	NA	NA	NA	NA	33.9	33.9	Alive	No
9	M	50	No	Parietal	Primary	Yes	cystic-solid	Yes	Non-GTR+RT+CT	-	11.5	-	unmethylated	-	-	20.0	26.5	Dead	Yes
10	F	29	No	Parietal-temporal	Secondary	Yes	solid	Yes	GTR+RT+CT	+	20	-	NA	-	NA	25.2	25.2	Alive	No
11	M	56	No	Temporal	Secondary	Yes	solid	Yes	GTR	NA	NA	NA	unmethylated	-	-	6.0	8.1	Dead	Yes
12	F	56	No	Frontal	Primary	Yes	solid	Yes	GTR+RT+CT	NA	10	NA	unmethylated	-	-	21.3	21.3	Alive	No
13	F	56	No	Temporal	Primary	Yes	solid	Yes	GTR+RT+CT	-	25	-	methylated	-	-	19.0	21.0	Alive	Yes
14	M	46	No	Temporal-occipital	Primary	Yes	solid	Yes	GTR+RT+CT	+	5	-	methylated	-	-	15.0	15.0	Alive	No
15	F	35	No	Frontal	Primary	Yes	solid	Yes	GTR+RT+CT	-	40	-	NA	NA	NA	14.3	14.3	Alive	No
16	M	20	Yes	Temporal	Primary	No	solid	No	GTR+RT+CT	+	30	-	unmethylated	-	-	12.9	12.9	Alive	No
17	M	6	No	Brainstem	Primary	No	Solid	Yes	Non-GTR+RT+CT	+	15	NA	NA	NA	NA	11.1	11.1	Alive	No
18	F	43	No	Frontal-parietal	Primary	Yes	cystic-solid	Yes	Non-GTR+RT+CT	-	15	-	unmethylated	-	-	9.4	9.4	Alive	No
19	F	13	Yes	Temporal	Primary	No	solid	Yes	GTR+RT+CT	NA	NA	NA	unmethylated	-	-	7.5	7.5	Alive	No
20	M	8	No	Temporal	Secondary	Yes	cystic-solid	Yes	GTR+RT+CT	-	15	-	unmethylated	-	-	7.4	7.4	Alive	No
21	M	52	No	Temporal	Primary	Yes	cystic-solid	Yes	Non-GTR+RT+CT	-	40	-	NA	-	+	6.9	6.9	Alive	No
22	M	53	Yes	Frontal	Primary	No	cystic-solid	Yes	GTR+RT+CT	-	10	-	unmethylated	-	+	6.7	6.7	Alive	No
23	F	42	No	CPA	Primary	Yes	solid	Yes	Non-GTR+RT+CT	+	20	-	NA	NA	NA	4.5	4.5	Alive	No
24	F	65	No	Temporal-parietal	Primary	Yes	cystic-solid	Yes	GTR+RT+CT	-	50	NA	methylated	-	+	1.5	1.5	Alive	No
25	F	71	Yes	Parietal	Primary	Yes	solid	Yes	GTR+RT+CT	-	50	NA	NA	NA	NA	0.9	0.9	Alive	No
26	M	27	No	occipital	Primary	Yes	solid	Yes	Non-GTR+RT+CT	+	30	-	unmethylated	-	-	0.5	0.5	Alive	No

M, male; F:female; GTR, gross total resection; Non-GTR, non- gross total resection; RT, radiotherapy; CT, chemotherapy; NA, not available; PFS, Progression-Free-Survival.

### Cases from literature

56 patients (29 males and 27 females) diagnosed of HGPXAs were included from January 1997 and October 2022. The mean age was 29.6 ± 19.6 years (range: 4-74 years). Preoperative seizure was observed in 20 (38.5%) patients. 45 patients experienced primary HGPXAs, 9 patients suffered malignant transformation of previous PXAs. The lesion locations include temporal lobe (n=27), frontal lobe (n=9), frontal-parietal lobe (n=5), parietal lobe (n=3), parietal-occipital lobe (n=2), cerebellum (n=2), lateral ventricle (n=2), tectal region (n=2), occipital lobe (n=1), occipital-parietal lobe (n=1), posterior fossa (n=1), brainstem (n=1). The morphology was classified as solid (n=19) and cystic-solid (n=16). MRI scans showed that peritumoral edema was significant in 23 (67.6%) patients and enhancement was observed in 32 (97%) patients. GTR and non-GTR were performed in 24 (44.4%) and 30 (55.6%) patients, respectively. Combined radiotherapy and chemotherapy was administered to 19 (38%) patients, radiotherapy alone was administrated to 12 (24%) patients, chemotherapy alone was administrated to 4 (8%) and 15 patients (30%) accepted no adjuvant therapy. 14 (53.8%, n=26) patients harbored BRAF V600E mutation and no patients (9 patients were available) harbored IDH mutations. BRAF inhibitors, such as dabrafenib, trametinib, were used for 7 patients who suffered tumor recurrence and showed clinical stability and radiographic improvement. It is worth noting that 10 (20.4%) patients experienced spinal metastasis. After a median follow-up of 31.4 ± 35.3 months (range: 0.75-144 months), the mortality and recurrence rates were 32.5% (13/40) and 70% (28/40), respectively (**Table 2**). A summary of the clinical characteristics of HGPXAs from both literature and our institute can be found in **Table 3**.

**Table 2:** Clinical and outcome review of reported 56 cases of APXAs from 1997 to 2022.

Author&Year	Age, Sex	Seizure	Site	Primary & Secondary	Peritumoral edema	Enhancement	Solid/cystic-solid	Treatment	BRAF	IDH	Ki-67/MIB-1 (%)	Treatment after Recurrence	PFS (months)	Follow-up (months)	Recurrence/metastasis	Outcome
Bayindir et al, 1997 (8)	9, F	Yes	Temporal	Secondary	No	Yes	cystic-solid	GTR+RT	NA	NA	31	S	6	10	Yes/Yes	Dead
Nasuha et al, 2003 (9)	10, M	Yes	Temporal	Primary	Yes	Yes	cystic-solid	Non-GTR+RT	NA	NA	NA	-	24	24	No	Alive
Lubansu et al, 2004 (10)	7, F	No	Temporal	Primary	Yes	Yes	cystic-solid	GTR+CT	NA	NA	<1	CT	1	26	Yes/Yes	Alive
Gelpi et al, 2005 (11)	43, F	No	Occipital	Primary	NA	NA	solid	GTR+RT	NA	NA	11.6	S	36	43	Yes	Alive
Asano et al, 2006 (12)	59, F	No	Temporal	Primary	Yes	Yes	solid	GTR	NA	NA	9.8	S	5	33	Yes	Dead
Baehring et al, 2006 (13)	23, F	No	Frontal	Primary	Yes	Yes	cystic-solid	GTR	NA	NA	NA	-	NA	NA	NA	NA
Chang et al, 2006 (14)	4, F	No	Cerebellum	Primary	No	Yes	cystic-solid	GTR+RT+CT	NA	NA	7.5	-	144	144	No	Alive
Koga et al, 2009 (1)	47, F	Yes	Frontal	Primary	Yes	Yes	solid	GTR	NA	NA	4	SRS, CT	16	66	Yes/Yes	Dead
Okazaki et al, 2009 (15)	5, M	No	Temporal	Primary	No	Yes	solid	Biopsy+CT	NA	NA	7.5	No	18	18	Yes/Yes	Alive
Fu et al, 2010 (16)	52, M	No	Lateral ventricle	Primary	Yes	Yes	cystic-solid	Non-GTR+RT+CT	NA	NA	8.7	-	6	6	No	Alive
Tsutsumi et al, 2010 (17)	16, F	No	Temporal	Primary	No	Yes	solid	Non-GTR+RT	NA	NA	6	S, RT	13	20	Yes	Alive
Rodríguez-Mena et al, 2012 (18)	54, M	No	Parietal-occipital	Secondary	Yes	Yes	cystic-solid	Biopsy	-	NA	10	No	0.75	0.75	Yes	Dead
Nern et al, 2012 (19)	57, M	No	Temporal	Primary	Yes	Yes	cystic-solid	GTR+RT	-	-	NA	S, RT, CT	10	28	Yes	Dead
Katayama et al, 2013 (20)	61, M	No	Tectal region	Primary	No	Yes	solid	Non-GTR+RT+CT	NA	-	32.7	-	10	10	No	Alive
Montano et al, 2013 (21)	22, M	Yes	Temporal	Primary	Yes	No	solid	GTR+RT+CT	NA	NA	4.5	-	12	12	No	Alive
Martinez et al, 2014 (22)	59, M	NA	Parietal	NA	NA	NA	NA	NA	-	-	12.5	NA	NA	NA	NA	NA
	22, M	NA	Temporal	NA	NA	NA	NA	NA	-	-	5	NA	NA	NA	NA	NA
Niamathullah et al, 2014 (23)	9, F	Yes	Frontal-parietal	Primary	Yes	Yes	cystic-solid	Non-GTR+RT	NA	NA	8	-	NA	NA	NA	NA
Benjamin et al, 2015 (24)	65, M	No	Temporal	Primary	No	Yes	cystic-solid	Non-GTR	-	NA	10	S, RT	0.5	4	Yes/Yes	Dead
Usubalieva et al, 2015 (25)	56, F	No	Brainstem	Primary	Yes	Yes	solid	GTR	-	NA	11	SRS	4	37	Yes	Alive
	35, F	No	Temporal	Primary	No	Yes	solid	Non-GTR +RT	+	NA	15.5	S, SRS, Dabrafenib	5	26	Yes	Dead
Choudry et al, 2016 (26)	55, M	Yes	Temporal	Primary	Yes	Yes	cystic-solid	GTR+RT	NA	NA	9	RT	2.25	4	Yes	Dead
Lee et al, 2016 (27)	41, M	NA	Temporal	Secondary	NA	NA	NA	Non-GTR+RT+CT	+	NA	20	Vemurafenib	6	15	Yes	Alive
Patibandla et al, 2016 (28)	35, M	No	Frontal	Primary	Yes	Yes	solid	Non-GTR +SR	NA	NA	NA	NA	NA	NA	NA	NA
Rutkowski et al, 2016 (29)	26, M	Yes	Temporal	Primary	NA	NA	NA	Non-GTR +RT+CT	NA	NA	NA	NA	NA	NA	Yes	Dead
	17, M	No	Frontal	Primary	NA	NA	NA	Non-GTR +RT+CT	NA	NA	NA	NA	NA	NA	Yes	Dead
	4, F	Yes	Temporal	Primary	NA	NA	NA	Non-GTR +CT	NA	NA	NA	NA	NA	NA	Yes/Yes	Alive
	4, F	No	Frontal	Secondary	NA	NA	NA	Non-GTR	NA	NA	NA	NA	NA	NA	Yes/Yes	Dead
	9, F	Yes	Temporal	Secondary	NA	NA	NA	Non-GTR	NA	NA	NA	NA	NA	NA	Yes	Alive
	38, F	Yes	Frontal	Secondary	NA	NA	NA	GTR	NA	NA	NA	NA	NA	NA	Yes	Alive
	74, F	No	Posterior fossa	Primary	NA	NA	NA	GTR	NA	NA	NA	NA	NA	NA	No	Alive
	54, M	No	Temporal	Primary	NA	NA	NA	Non-GTR+RT+CT	NA	NA	NA	NA	NA	NA	Yes	Alive
Suzuki et al, 2016 (30)	17, M	No	Tectal region	Primary	No	Yes	solid	GTR+RT+CT	-	NA	10	-	24	24	No	Alive
Yamada et al, 2016 (31)	42, M	No	Temporal	Primary	Yes	Yes	cystic-solid	Non-GTR+RT+CT	NA	NA	20	No	5.6	5.8	Yes	Dead
Brown et al, 2017 (32)	21, F	No	Temporal	Primary	Yes	Yes	cystic-solid	GTR+RT	+	NA	NA	S, Dabrafenib, Vemurafenib	3	19	Yes	Alive
Migliorini et al, 2017 (33)	32, F	NA	Parietal	Secondary	No	Yes	solid	Non-GTR+RT+CT	+	NA	NA	Dabrafenib, Trametinib	6	11	No	Alive
Thara et al, 2017 (34)	42, M	Yes	Temporal	Primary	Yes	NA	solid	Non-GTR	+	NA	8.5	NA	NA	NA	NA	NA
Amayiri et al, 2018 (35)	16, F	Yes	Parietal	Primary	Yes	Yes	cystic-solid	Non-GTR+RT+CT	+	NA	NA	S, CT, Dabrafenib	36	66	Yes	Alive
Hussain et al, 2018 (36)	43, M	No	Frontal	Primary	NA	NA	NA	GTR+RT	+	NA	NA	Vemurafenib, Trametinib	24	34	Yes	Alive
Oladiran et al, 2018 (37)	28, M	No	Temporal	Primary	Yes	Yes	solid	Non-GTR	+	NA	NA	-	NA	NA	NA	NA
Pradhan et al, 2018 (38)	11, M	No	Occipital-parietal	Primary	NA	NA	NA	Non-GTR+NA	NA	NA	6	-	12	12	No	Alive
	40, F	No	Temporal	Primary	NA	NA	NA	GTR+NA	NA	NA	12.5	-	12	12	No	Alive
	19, M	Yes	Frontal-parietal	Primary	NA	NA	NA	Non-GTR+NA	NA	NA	20	-	12	12	No	Alive
	18, F	No	Frontal-parietal	Primary	NA	NA	NA	Non-GTR+NA	NA	NA	18	Biopsy	12	36	Yes/Yes	Alive
Roberti et al, 2018 (39)	65, F	No	Lateral ventricle	Primary	No	Yes	solid	GTR	NA	NA	2	No	3	3	Yes	Alive
Saraf et al, 2018 (40)	5, M	Yes	Temporal	Primary	Yes	Yes	solid	Non-GTR+GN+CT	-	NA	25	CT, Everolimus	NA	60	Yes	Alive
Fukushima et al, 2019 (41)	15, F	Yes	Temporal	Secondary	Yes	Yes	solid	GTR	+	NA	14.4	Multimodal therapies	9	20	Yes	Dead
Nakamura et al, 2019 (42)	14, F	No	Cerebellum	Primary	NA	NA	NA	Non-GTR+RT+CT	-	-	NA	CT	NA	9.4	Yes	Dead
Purkait et al, 2019 (43)	35, F	No	Parietal-occipital	Primary	Yes	Yes	cystic-solid	GTR+RT	+	-	12	No	18	18	Yes	Alive
Sasaki et al, 2019 (44)	12, M	No	Frontal	Primary	Yes	Yes	cystic-solid	Non-GTR	-	-	NA	S, RT, CT	2	12	Yes	Alive
Thomas et al, 2019 (45)	16, F	Yes	Frontal	Primary	NA	NA	NA	Non-GTR+RT+CT	+	NA	15	Dabrafenib, Trametinib	6	23	Yes/Yes	Dead
Liu et al, 2020 (46)	28, M	Yes	Frontal-parietal	Primary	Yes	Yes	solid	Non-GTR+CT	-	-	15	-	NA	NA	No	Alive
Matsumoto et al, 2020 (7)	32, M	Yes	Temporal	Secondary	No	Yes	solid	GTR+RT+CT	+	-	12.1	CT	24	29	Yes/Yes	Dead
Ronsley et al, 2020 (47)	12, M	No	Temporal	Primary	NA	NA	NA	GTR+ RT+CT	+	NA	NA	GTR+CT	2	144	Yes	Alive
	12, M	Yes	Frontal-parietal	Primary	NA	NA	NA	GTR+RT+CT	-	NA	NA	-	132	132	No	Alive
	12, F	Yes	Temporal	Primary	NA	NA	NA	GTR+RT+CT	+	NA	NA	-	48	48	No	Alive

S, surgery, NA, not available.

**Table 3:** Summary of clinical characteristics of Anaplastic pleomorphic xanthoastrocytoma from literature and our institute.

Variable	Prior studies (n=56)		Our series	Overall
	No. of available cases	Value		
Mean age, years	56	29.6 ± 19.6	36.7 ± 20.3	31.9 ± 20.0
Sex (M/F)	56	29/27	11/15	40/42
Seizure	52	20 (38.5)	8 (30.8)	28 (35.9)
Temporal	56	27 (48.2)	13 (50)	40 (48.8)
Primary/Secondary	54	45/9	22/4	67/13
Peritumoral edema	34	23 (67.6)	21 (80.8)	44 (73.3)
Solid/cystic-solid	35	19/16	16/10	35/26
Enhancement	33	32 (97.0)	25 (96.2)	57 (96.6)
BRAF	26	14 (53.8)	6 (40)	20 (48.8)
GTR	54	24 (44.4)	17 (65.4)	41 (51.3)
RT	50	31 (62)	22 (84.6)	53 (69.7)
CT	50	23 (46)	20 (76.9)	43 (56.6)
BRAF inhibitor	56	7 (12.5)	0	7 (8.5)
Recurrence	49	35 (71.4)	7 (26.9)	42 (46)
Death	49	16 (32.7)	6 (23.1)	22 (29.3)
Mean PFS, months	34	19.5 ± 32.2	19.0 ± 21.2	19.3 ± 27.8
Mean FU, months	40	31.4 ± 35.3	20.5 ± 21.2	27.1± 30.8

### Pooled analysis

**Figure 1:**
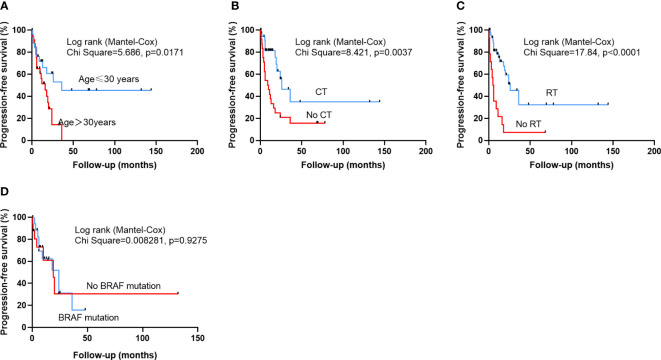
Kaplan-Meier survival curves illustrating the difference PFS. Patients older than 30 years **(A)**, who did not undergo CT **(B)**, or had no RT **(C)** had a significantly worse PFS. There is no significantly statistical difference between BRAF mutation group and no BRAF mutation group in PFS **(D)**.

**Figure 2:**
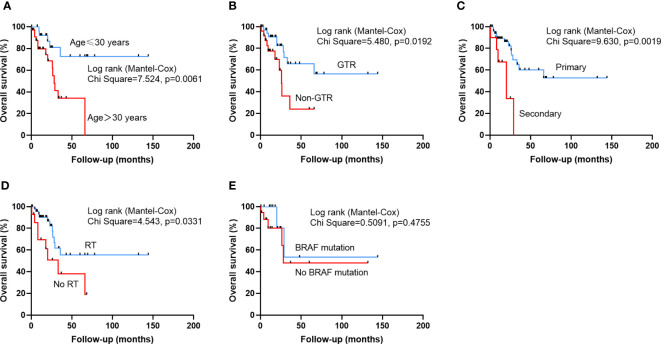
Kaplan-Meier survival curves illustrating the difference OS. Patients older than 30 years **(A)**, who did not undergo GTR **(B)**, secondary HGPXAs, or had no RT **(C)** had a significantly worse OS. There is no significantly statistical difference between BRAF mutation group and no BRAF mutation group in OS **(E)**.

The mean PFS time was 19.3 ± 27.8 months, and the 1-, 2-year and 5-year recurrence rates were 61.1%, 38.7%, 26.1%, respectively. The mean OS time was 27.1 ± 30.8 months, and the 1-, 2-year and 5-year OS rates were 85.3%, 74.2% and 51.5%, respectively. The univariate cox regression analysis revealed that no RT (HR 4.105, 95% CI 1.998-8.432, p<0.001), no CT (HR 2.724, 95% CI 1.329-5.585, p=0.006), age>30 years (HR 2.436, 95% CI 1.135-5.226, p=0.022) years predicted a poor PFS. Multivariate cox regression analysis confirmed that non-GTR (HR 2.633,9 5% CI 1.203-5.762, p=0.015), age>30 years (HR 2.620, 95% CI 1.183-5.804, p=0.018), no CT (HR 2.371, 95% CI, 1.034-5.437, p=0.042) and no RT (HR 2.995, 95% CI 1.344-6.673, p=0.007) were significantly associated with poorer PFS (**Table 4**). The univariate cox regression analysis revealed that age>30 years (HR 3.946, 95% CI 1.378-11.306, p=0.011), non-GTR (HR 2.876, 95% CI 1.142-7.254, p=0.025), secondary HGPXAs (HR 4.494, 95% CI 1.589-12.708, p=0.005) and no RT (HR 2.593, 95% CI 1.042-6.453, p=0.041) predicted a poor OS. Multivariate cox regression analysis confirmed that non-GTR (HR 7.963, 95% CI 2.368-26.776, p=0.001), secondary HGPXAs (HR 7.567, 95% CI 2.221-25.781, p=0.001), age≥30 (HR 3.568, 95% CI 1.190-10.694, p=0.023) and no RT (HR 4.490,95% CI 1.469-13.726, p=0.008) predicted a poor OS (**Table 5**). Kaplan-Meier analysis showed that age>30 years (p=0.0171), no CT (p=0.0037) and no RT (p<0.0001) predicted a poor PFS (**Figures 1A-C**). Age>30 years (p=0.0061), non-GTR (p=0.0192), secondary HGPXAs (p=0.0019) and no RT (p=0.0331) predicted a poor OS (**Figures 2A-D**). There is no significant statistical difference between BRAF mutation group and no BRAF mutation for PFS (**Figure 1D**) and OS (**Figure 2E**).

**Table 4:** Cox regression model for risk factors predicting PFS.

Variable	Number of patients	Recurrence	Univariate Analysis		Multivariate Analysis	
			HR (95% CI)	P value	HR (95% CI)	P value
Overall	60	32 (53.3)				
Age						
Age ≤ 30	26	11 (34.4)	Reference		Reference	
Age>30	34	21 (65.6)	2.436 (1.135-5.226)	0.022^*^	2.620 (1.183-5.804)	0.018^*^
Sex						
Female	32	17 (53.1)	Reference			
Male	28	15 (53.6)	1.259 (0.620-2.556)	0.524		
Primary						
Yes	50	25 (50)	Reference			
No	10	7 (70)	1.769 (0.750-4.173)	0.193		
Location						
Others	29	13 (44.8)	Reference			
Temporal	31	19 (61.3)	1.724 (0.846-3.511)	0.134		
GTR						
Yes	23	14 (60.9)	Reference		Reference	
No	37	18 (48.6)	1.829 (0.897-3.729)	0.097	2.633 (1.203-5.762)	0.015^*^
RT						
Yes	46	19 (41.3)	Reference		Reference	
No	14	13 (92.9)	4.105 (1.998-8.432)	<0.001^*^	2.995 (1.344-6.673)	0.007^*^
CT						
Yes	36	12 (33.3)	Reference		Reference	
No	24	20 (83.3)	2.724 (1.329-5.585)	0.006^*^	2.371 (1.034-5.437)	0.042^*^

*: p< 0.05 was considered statistically significant.

**Table 5:** Cox regression model for risk factors predicting OS.

Variable	Number of patients	Death	Univariate Analysis		Multivariate Analysis	
			HR (95% CI)	P value	HR (95% CI)	P value
Overall	62	19 (30.6)				
Age						
Age ≤ 30	28	5 (17.9)	Reference		Reference	
Age>30	34	14 (41.2)	3.946 (1.378-11.306)	0.011^*^	3.568 (1.190-10.694)	0.023^*^
Sex						
Female	33	9 (27.3)	Reference			
Male	29	10 (34.5)	1.762 (0.710-4.370)	0.222		
Primary						
Yes	52	13 (25)	Reference		Reference	
No	10	6 (60)	4.494 (1.589-12.708)	0.005^*^	7.567 (2.221-25.781)	0.001^*^
Location						
Others	30	7 (23.3)	Reference			
Temporal	32	12 (37.5)	1.855 (0.725-4.472)	0.197		
GTR						
Yes	25	10 (40)	Reference		Reference	
No	37	9 (24.3)	2.876 (1.142-7.254)	0.025^*^	7.963 (2.368-26.776)	0.001^*^
RT						
Yes	14	8 (57.1)	Reference		Reference	
No	48	11 (22.9)	2.593 (1.042-6.453)	0.041^*^	4.490 (1.469-13.726)	0.008^*^
CT						
Yes	24	12 (50)	Reference			
No	38	7 (18.4)	2.139 (0.839-5.451)	0.111		

*: p< 0.05 was considered statistically significant.

## Discussion

Pleomorphic xanthoastrocytoma (PXA) is a rare brain tumor, which was first reported in 1979 (48, 49). ‘PXA with anaplastic features’ are labelled as anaplastic pleomorphic xanthoastrocytoma (APXA), WHO grade III, according to the 2016 World Health Organization Classification of Tumors of the Central Nervous System (3). According recent World Health Organization Classification of Tumors of the Central Nervous System, PXA was classified into WHO grade 2 or WHO grade 3 tumors (5). Most of the information available about APXA comes from isolated case reports. In our institute, 200 cases with PXAs were pathologically confirmed from August 2014 and September 2021. Among them 26 cases were grade 3 PXAs and 174 cases were grade 2 PXAs, HGPXAs are estimated to comprise 13% of all PXAs in our single center.

Mallick et al. reported the median age of grade 2 PXA patients was 20 years *via* analyzing 167 cases and Rodrigues et al. reported the median age of grade 2 PXA patients was 21 years *via* analyzing 346 cases (49, 50). In our pooled cohort, HGPXAs tended to affect middle-aged patients with a median age of 30.5 years, compared to their grade 2 PXA patients. We also found that age>33 years was a risk factor for PFS (p=0.018) and OS (p=0.023) *via* multivariate cox regression analysis. There is no gender predominance in HGPXAs in this study, which is consistent with previous studies (50). Most intracranial HGPXAs were located in supratentorial area and temporal lobe was the first commonest region, only 5 cases located in infratentorial area. Preoperative seizure is common with the occurrence of 35.9%. 8 cases from our institute presented with preoperative seizure were free of seizure after GTR. HGPXAs can be divided into primary tumors or secondary tumors. Of the pooled analysis, 67 cases were primary tumors and 13 cases were secondary tumors, and secondary tumors had a poorer OS than primary tumors (p=0.001). Radiologically, HGPXAs often appear T1 isointense and T2 hyperintense and the cystic component is often hypodense On Magnetic Resonance Imaging (MRI). PXA usually contains solid and cystic components and the solid component often enhances (8, 51). She et al. reviewed MR imaging features of 9 patients with APXA and 10 patients with PXA. The presence of heterogeneous enhancement of solid mass was observed more frequently in patients with APXA than in those with PXA (52). In our study, enhancement was observed in almost all HGPXAs patients (96.6%) and solid tumors are more common than cystic-solid tumors (35 VS 26 cases). 73.3% patients with HGPXAs had obvious peritumoral edema. However, through cox regression analysis, no peritumoral edema could not predict a better PFS or OS.

Due to the rarity of HGPXAs, there is limited research to identify risk factors for OS or PFS. Patibandla et al. pointed that a complete surgical excision is required for a prolonged disease-free interval (28). Rodrigues et al. analyzed 62 cases of APXA from SEER database and concluded that complete surgical resection could not bring improved outcomes. In our study, we found non-GTR group was associated with a poor PFS (p=0.015) and OS (p=0.001) than GTR group. Maximum safe resection, if feasible, should be the first goal of the neurosurgeon. The role of postoperative radiotherapy and chemotherapy for APXAs is still uncertain (1). Marton et al. reviewed 9 cases with APXA underwent conventional fractionated radiotherapy, but the effect of treatment is not significant (53). Koga et al. reported a case of APXA treated with postoperative stereotactic irradiation (STI) that resulted in long-term control of the tumor (1). Postoperative chemotherapy has been commonly considered ineffective for the treatment of PXAs (54). In our pooled analysis, we found that no postoperative radiotherapy group was associated with a poor PFS (p=0.007) and OS (p=0.008) than postoperative radiotherapy group and no postoperative chemotherapy can predict a better PFS (p=0.042). Based on these, we recommended that postoperative radiotherapy and chemotherapy should be added to conventional treatment protocols. Molecular markers are increasingly used to help doctors to diagnose or subclassify gliomas. BRAF mutations were found in 70% PXAs, but involved less common in HGPXAs (55). Phillips et al (56) performed comprehensive genomic profiling on 15 cases with APXA and found 5 cases with TERT promoter hotspot mutation. In our study, BRAF p.V600E mutations were detected in 48.8% of patients (20/41), and TERT promoter mutations were found in 23.1% of patients (3/13). It is reported that compared to the BRAF wild-type PXA, BRAF-mutated PXA revealed prolonged survival (6). However, we did not find any relationship between BRAF mutation and PFS (p=0.9275) or OS (p=0.4755) for HGPXAs. In previous reports, small molecule drugs, such as BRAF, MEK inhibitors were used for some patients. Brown et al. reported a 21-year-old female suffered occurrence of APXA with a BRAFV600 mutation following 2 operations and radiotherapy. Then she was orally administered with BRAF inhibitors. She was well up to the last follow-up (32). Hussain et al. reported a 43-year-old male underwent GTR plus radiotherapy of an APXA, but, unfortunately, the tumor progression happened 2 years later. Because the tumor harbored a BRAFV600 mutation, combination of vemurafenib (BRAF inhibitor) and trametinib (MEK inhibitor) was added to treatment strategy. Clinical stability and radiographic improvement were achieved for 6 months after the targeted therapy (36). Targeted therapy may bring hope to HGPXAs patients, but more data is needed to prove its effectiveness.

According to previous studies, OS of PXA is favorable with 5-year survival rates of >75% and PFS rates at 5 years are >60% (4, 57). Patibandla et al (28) reviewed 17 cases of APXA reported in the literature till 2010 and found that among 13 patients whose follow-up information was available, 8 patients died with the mean overall survival was 26.1 months (ranges from 1 to 66 months). Compared to PXA, we found that HGPXAs have a relatively poor clinical outcomes with 5-year OS of 51.5% and 5-year PFS of 26.1% and 19 patients died with a mean overall survival was 19.8 months (ranges from 0.75 to 66 months). Although no tumor metastasis was found in our institute, 13.3% (10/75) patients experienced spinal metastasis were founded in reported cases. Spinal MRI should be taken into account during postoperative follow-up for early treatment of the metastatic tumor.

## Conclusion

High grade pleomorphic xanthoastrocytomas are very rare brain tumors. Children and younger adults have better clinical outcome than elderly patients. Secondary HGPXAs had worse OS than primary HGPXAs. Complete surgical excision plus RT and CT is recommended for this entity. The frequency of BRAF mutations in HGPXAs is 47.5% (19/40) in this study, however, we do not find the connections between BRAF mutations and clinical outcomes. Future studies with larger cohorts are necessary to verify our findings.

## Data availability statement

The raw data supporting the conclusions of this article will be made available by the authors, without undue reservation.

## Ethics statement

The studies involving human participants were reviewed and approved by human research ethics committee of Beijing Tiantan Hospital. The patients/participants provided their written informed consent to participate in this study.

## Author contributions

Writing, original draft, Conceptualization: PZ; Statistical analysis: TS, WW; Literature review: TL, YW; Follow up: MZ; Methodology, Resources: ZW, JZ; Review, editing, Supervision: LZ. All authors contributed to the article and approved the submitted version.
